# Direct Electrophoretic Deposition and Characterization of Thin-Film Membranes Based on Doped BaCeO_3_ and CeO_2_ for Anode-Supported Solid Oxide Fuel Cells

**DOI:** 10.3390/membranes12070682

**Published:** 2022-06-30

**Authors:** Elena Pikalova, Denis Osinkin, Elena Kalinina

**Affiliations:** 1 Laboratory of Solid Oxide Fuel Cells, Institute of High Temperature Electrochemistry, Ural Branch of the Russian Academy of Sciences, Yekaterinburg 620137, Russia; e.pikalova@list.ru (E.P.); osinkinda@mail.ru (D.O.); 2 Department of Environmental Economics, Graduate School of Economics and Management, Ural Federal University, Yekaterinburg 620002, Russia; 3 Laboratory of Complex Electrophysic Investigations, Institute of Electrophysics, Ural Branch of the Russian Academy of Sciences, Yekaterinburg 620016, Russia; 4 Department of Physical and Inorganic Chemistry, Institute of Natural Sciences and Mathematics, Ural Federal University, Yekaterinburg 620002, Russia

**Keywords:** electrophoretic deposition, solid oxide fuel cell, thin-film electrolyte membrane, anode-supported cell, doped CeO_2_, doped BaCeO_3_, deposition kinetics, sintering kinetics, densification

## Abstract

In this work, a technology was developed for the formation of BaCe_0.8_Sm_0.2_O_3_+1 wt% CuO (BCS-CuO)/Ce_0.8_Sm_0.2_O_1.9_ (SDC) thin-film electrolyte membranes for intermediate-temperature solid oxide fuel cells (IT-SOFCs) on porous NiO-BCS-CuO anode substrates using direct electrophoretic deposition (EPD). The effect of increasing the zeta potential when modifying the base suspension of a micro-sized SDC-gn powder (glycine–nitrate method) with the addition of a SDC-lec nanopowder (laser evaporation–condensation) was investigated. Dependences of the current strength on the deposition time and the deposited weight on the EPD voltage were obtained, and evolution of the morphology of the coatings during the modification of the SDC-gn suspension and a suspension of BCS-CuO powder was studied. The compatibility of the shrinkage kinetics of the SDC, the BCS-CuO electrolyte coatings and the NiO-BCS-CuO anode substrate was studied during the high-temperature sintering. Dense BCS-CuO/SDC films of different thicknesses were obtained for the first time on porous NiO-BCS-CuO anode substrates and comprehensive microstructural and electrochemical studies were carried out. The developed technology can be applied to the formation of anode-supported SOFCs with thin-film electrolyte membranes.

## 1. Introduction

Electrochemical energy devices such as solid oxide fuel cells (SOFCs) have attracted many efforts of scientific groups and manufacturers for commercialization due to their extraordinary power efficiency, fuel flexibility and environmental friendliness [1]. An established trend to decreasing the SOFCs working temperature is a reliable way to reducing the production cost through the using cheaper functional materials and extending the service life of SOFC-based energy systems [2]. Unfortunately, decreased temperatures lead to increasing the resistivity of all thermo-activated processes in SOFCs. The key challenge in the realization of the reduced-temperature approach is to maintain low ohmic resistance of the electrolyte membrane. Thus, traditional Yttria doped Zirconia electrolytes were replaced by new materials possessing superior conductivity in the intermediate-temperature (IT) range (600–800 °C) [3]. These are materials that have pure ionic conductivity [4,5], proton conductivity [6,7] and mixed ionic–electronic (MIEC conductivity) [8,9], as well as nanocomposite electrolyte materials [8,9,10]. 

One of the common approaches for decreasing the SOFCs’ electrolyte ohmic resistance is a decrease of this thickness to an optimum value [11]. In this sense, an electrode-supported design is currently the most widely used geometry for the fabrication of IT-SOFCs. The technical challenge in this design involves depositing crack-free, gas-tight electrolyte layers with a thickness of 1 (or even less) to 50 μm on the electrode (preferable anode) substrates of high porosity (50% or even more). The problems that appear in the process of the electrode-supported cells’ formation include delamination at the electrode–electrolyte interfaces and microstructure degradation of the electrode support during electrolyte film sintering, causing difficulties in gas transportation within the thick electrode layer. To reduce these drawbacks, structural optimizations have been proposed for both anode [12,13,14,15] and cathode supports [16,17]. 

Naturally, many methods have been developed to produce gas-tight electrolyte layers on porous substrates [18,19,20]. One of the cost-effective and technologically simple wet processes is electrophoretic deposition (EPD), which has been widely studied for the fabrication of various electrolyte films [20,21]. It is characterized by a short deposition time (approximately 1–10 μm per 1 min) and flexibility to the dispersity of the powders used for the suspension preparation (from micro- to nano-sized), as well as low requirements for the shape and porosity of the supporting substrates. Moreover, EPD allows direct deposition on non-conducting substrates of high porosity [22]. This method has been used for the EPD of YSZ films on porous NiO-YSZ anodes [23,24,25,26,27]. Being saturated with a solvent, continuous pores in the substrate with a minimum threshold porosity of more than 50% have been shown to form a “conductive path” between the electrode and particles in the suspension, allowing dense film to be formed [24]. 

Gadolinia and samaria-doped ceria electrolytes (GDC and SDC, respectively) are known as the most prospective candidates for IT-SOFCs because of their superior ionic conductivity, which exceeds that of YSZ by nearly 6–7 times [8]. Moreover, these electrolytes demonstrate excellent compatibility with high-performance cathode materials suitable for IT-SOFCs [28,29,30]. However, one main limitation of the CeO_2_-based electrolytes is the appearance of n-type electronic conductivity caused by Ce^4+^ to Ce^3+^ under reducing conditions in the SOFC anode channel. This results in decreasing the cells’ Nernstian potential and maximum power output [8]. The electrolyte bilayering strategy, which was supposed to introduce a unipolar ionic conductor layer on the anode side as a barrier against ceria reduction, has been successfully applied to improve the performance of IT-SOFCs based on doped CeO_2_ [31]. The method of a direct layer-by-layer EPD has been applied for the formation of a bilayer YSZ/Sm_2_O_3_-doped CeO_2_ (SDC) electrolyte film on a porous NiO–YSZ anode substrate [32] followed by its co-sintering with the substrate. As EPD electrolyte coatings require high sintering temperatures, a mismatch in the coefficient of the thermal expansion values of the layers within the multilayer structure may cause their delamination, as well as curvature of the substrate during sintering [33]. To avoid delamination, in [32] the thickness of the SDC film deposited over the YSZ film of 4 μm in thickness was reduced to ca. 1 μm. Moreover, high sintering temperatures of YSZ/SDC films can cause their chemical interaction, thus decreasing the conducting properties [34]. Thus, to keep the thickness of a CeO_2_-based film as the main electrolyte on the required level and avoid delamination and interaction with a barrier layer (on the anode or cathode side), it is of great importance to find compatible materials for bilayer films to replace traditionally used YSZ. 

Electrolytes based on doped BaCeO_3_ are known to be suitable for SOFCs with decreased operating temperatures due to their low activation energy for proton conduction [35]. For example, Sm-doped BaCeO_3_ possesses the conductivity of 8–10 mScm^−1^ at 600 °C in wet air [36]. Cu-doping significantly increases the sinterability of proton-conducting ceramics [37]. Moreover, added in a small amount as a sintering additive, CuO can improve not only sinterability but also the grain boundary (GB) conductivity of doped BaCeO_3_, lowering the GB density [38]. Recently, we demonstrated good compatibility of electrophoretically deposited Cu-doped BaCe_0.8_Sm_0.2_O_3_ barrier layers (~20 μm) with a dense SDC substrate (550 μm) [39]. 

In the present work, for the first time the formation of bilayer Sm-doped BaCeO_3_/Sm-doped CeO_2_ electrolyte membranes with various layer thicknesses was performed on porous anode substrates by direct EPD. To increase sinterability, a Sm-doped BaCeO_3_ electrolyte was modified by the addition of 1 wt% CuO (BCS-CuO). The investigation included the study of the kinetic properties of the electrolyte suspensions. Particularly, the composite effect of increasing the zeta potential was studied when modifying the base suspension of micro-sized SDC-gn powder, obtained via nitrate combustion synthesis, by the addition of SDC-lec nano-sized powder obtained by the laser evaporation–condensation (LEC) method. The effects of the EPD modes on the deposition process, i.e., the change in the current strength on the deposition time and the deposition weight as well as the morphology of the coatings on the EPD voltage, were scrutinized. To establish favorable sintering conditions, a comparative study of the shrinkage kinetics of the SDC and BCS-CuO electrolyte coatings and the 50 wt% NiO–50 wt% BCS-CuO (NiO-BCS-CuO) anode substrate was carried out. Finally, sintered dense BCS-CuO/SDC coatings on the porous NiO-BCS-CuO anode substrates were formed and electrochemical tests of the cells were carried out with interpretation of the obtained impedance spectroscopy data using analysis of the distribution of relaxation times (DRT) [40,41]. 

## 2. Materials and Methods

### 2.1. Synthesis and Characterization of the Electrolytes 

Micro-sized Ce_0.8_Sm_0.2_O_1.9_ (SDC-gn) electrolyte powder was synthesized via nitrate combustion using Ce(NO_3_)_3_⋅6H_2_O (99.9% wt) and Sm(NO_3_)_3_⋅6H_2_O (99.0% wt) as starting materials, as well as citric acid (cit) as a chelating agent and glycine (gl) as an organic fuel in the amount of 0.6_gl_:0.8_cit_ per 1 mole of the mixed oxide. All reagents were mixed with the addition of a small amount of distilled water until complete dissolution. Then the mixture was heated until the formation of the xerogel with subsequent self-ignition. The resulting fluffy powder of yellow color was calcined in air at 900 °C for 5 h. After calcination, the powder was milled in ethanol in a planetary mill Pulverisette 7 (Retsch, Hann, Germany) for 1 h using zirconia grinding media and a plastic vial. The specific surface area of the powder, S_BET_, was determined using a SORBI N 4.1 Instrument (Meta, Russia). Calibration of the instrument was performed using the Al_2_O_3_ standard sample with the surface area of 211 m^2^/g (δ = ±1.8%).

Nano-sized SDC-lec electrolyte powder was obtained via laser evaporation–condensation [42]. The powder for the formation of the ceramic target used for further evaporation was obtained from the starting materials of CeO_2_ (99.9% wt) and Sm_2_O_3_ (99.9% wt) via a solid-state reaction method. An appropriate ratio of the starting materials was mixed with ethanol in the planetary mill. The synthesis involved two steps at temperatures of 900 °C, 10 h at 1050 °C and 10 h with intermediate milling for 1 h. The target was produced by pressing the as-prepared powder at 300 MPa into a disk with 8 cm in diameter and 2 cm in height followed by sintering at 1300 °C for 3 h. The ceramic target was evaporated using the ytterbium laser radiation sources LS-06 with a fiber transmission system (IPG Photonics, Oxford, MA, USA) at an effective output power of 600 W followed by condensation in a cyclone. 

Micro-sized BaCe_0.8_Sm_0.2_O_3_ (BCS-CuO) powder was obtained via nitrate combustion using BaCO_3_ (99.2% wt), Ce(NO_3_)_3_∙6H_2_O (99.9% wt) and Sm(NO_3_)_3_·6H_2_O (99.0% wt) as starting materials. Modification of the powder by the addition of CuO as a sintering additive directly during the synthesis was proposed earlier in [43]. Thus, CuO (99.0% wt) taken in an amount of 1 wt% and BaCO_3_ powders were dissolved in a minimum amount of diluted HNO_3_ to obtain the corresponding nitrates and then mixed with the rest of the reagents in a small amount of distilled water. Citric acid and glycerin (glyc) were introduced into the mixture in the ratio of 1.5_glyc_:0.5_cit_ per 1 mole of the mixed oxide. After complete dissolution of the reagents under heating up to 100 °C and constant stirring, 10 % ammonia solution was added to establish the pH value at 6–7. Then the solution was evaporated at 200 °C until self-ignition. The obtained powder was fired at 700 °C for 1 h to eliminate organic residues and then calcined twice at the temperature of 1050 °C for 5 h and 1150 °C for 5 h with intermediate milling. After the final synthesis the powder used for the suspension preparation was milled for 3 h in ethanol in the planetary mill using zirconia grinding media and the plastic vial.

The XRD analysis of the obtained powders was performed using an XRD-7000 diffractometer (Shimadzu, Kyoto, Japan) with Bragg–Brentano focusing at the power of 40 kV and 30 mA in a CuKα radiation, in a 2θ angle range of 25–80° with a step of 0.02° and a fixed time of 5 s at each point during the powder characterization and 3 s in the surface studies. Before the measurement, calibration of the X-ray diffraction instrument was carried out using the internal Si standard. Data processing and phase identification were performed using the PDF-2 database (ICDD, Newtown Square, PA, USA).

### 2.2. Fabrication of the Anode Substrates and Their Characterization 

For the fabrication of anode substrates, the BCS-CuO electrolyte powder was calcined at 1100 °C for 5 h to decrease its specific surface area down to 1.2 m^2^/g. NiO (98.4% wt) and the electrolyte powder taken in a ratio of 50/50 wt% were mixed with starch in an amount of 10 wt% in the ethanol medium in the planetary mill for 1 h using iron grinding media and an agate vial. The powder was dried and then compacted by uniaxial semi-dry pressing at a pressure of 300 MPa with addition of polyvinyl butyral binder into the disks of 15 mm in diameter. The as-prepared substrates were sintered at a temperature of 1200 °C for 4 h in air at a heating/cooling rate of 100 °C/h (Nabertherm LHT-04/18 furnace, Lilienthal, Germany). Pre-sintered anode substrates were polished using a diamond grinding disk and then treated in an ultrasonic bath in isopropyl alcohol to clean the surface and annealed at 900 °C for 1 h. The density of the anode samples was determined by weighing and measuring the geometric dimensions and amounted to approximately 59%. 

Dilatometric measurements were carried out using a DIL 402 C dilatometer (NETZSCH, Selb, Germany) in the heating mode up to 1500 °C and under subsequent cooling at a constant rate of 5 °C/min on the samples of the anode and electrolyte materials compacted by uniaxial pressing at 1.2 GPa using a magnetic-pulse press (IEP UB RAS, Ekaterinburg, Russia), as described elsewhere [44]. The sample of the anode material was heated up to 1200 °C to burn out the pore former and then cooled down to room temperature. The experiments were carried out in an air flow of 100 mL/min.

### 2.3. Preparation of the Suspensions Based on the Electrolyte Materials and Their Characterization

Taking into account the results of our previous study [39], a mixed dispersion medium of isopropanol/acetylacetone in a ratio of 70/30 vol.% was chosen for the preparation of the suspensions based on the electrolyte materials. Suspensions of SDC-gn + 5 wt% SDC-lec and BCS-CuO with a concentration of 10 g/L were prepared by weighing the powders and then sonication using a UZV-13/150-TH ultrasonic bath with a generator power of 210 W at the operating frequency of 22 kHz for 5–125 min (at 25 °C). The temperature was controlled with a thermometer and maintained at a given level by the water exchange in the ultrasonic bath. To facilitate deposition, molecular iodine was added to the BCS-CuO suspension in an amount of 0.4 g/L. The electrokinetic zeta potential and pH in the as-prepared suspensions were measured by the electroacoustic method using a DT-300 analyzer (Dispersion Technology, Bedford Hills, NY, USA). 

### 2.4. Electrophoretic Deposition of the Thin-film Electrolyte Layers 

Electrophoretic deposition of SDC-gn + 5 wt% SDC-lec and BCS-CuO electrolyte layers was performed using a specialized software-controlled installation (IEP UB RAS, Russia) in a constant voltage mode. The current strength during the deposition was measured using an Intelligent Digital Multimeter UNI-T UT71E (Uni-Trend Technology, Dongguan, China). For the deposition of the electrolyte layers an anode substrate was placed on the cathode of the EPD cell. A stainless-steel disk of 12 mm in diameter was used as a counter electrode of the EPD cell. The distance between the electrodes in the EPD cell was fixed at 10 mm. The details of the EPD process of the electrolyte films and their sintering are given below in Section 3.4. 

The surface morphology of the deposited layers was controlled using an ST-VS-520 optical microscope (STAT, Ekaterinburg, Russia). The thickness of the deposited green coatings was estimated from the weight of the film, the film surface area and the theoretical density of the deposited material. The actual thickness of the sintered coating was determined from the cross-sectional SEM images obtained using a JSM-6390 LA scanning electron microscope (JEOL, Tokyo, Japan) equipped with an energy-dispersion X-ray (EDX) microanalysis system. SEM images in the back-scattered electron (BSE) and secondary electron (SE) modes were obtained at a high voltage of 10 kV. The morphology of the micro-sized powders used for the suspension preparation was examined using the same electron microscopy equipment. Electron micrographs of the SDC-lec nano-sized powder were obtained using a JEM 2100 transmission electron microscope (JEOL, Tokyo, Japan). 

### 2.5. Fuel-Cell Fabrication and High-temperature Investigations

For the electrochemical characterizations, anode-supported half-cells with bilayer membranes based on doped BaCeO_3_ (BCS) and CeO_2_ (SDC) produced using the EPD method (Section 3.4) were used. On the SDC side of the sintered half-cells a fuel cell cathode was fabricated from a platinum paste. The area of the supporting anode was about 1.2 cm^2^, the area of the cathode was about 0.3 cm^2^. Current leads were made of a platinum wire of 0.2 mm in diameter, fixed on the Pt cathode with the paste and sintered at 1000 °C for 1 h. Next, a mounting ceramic ring made of YSZ electrolyte was placed on the side of the SDC electrolyte and sintered using high-temperature glass to the SDC electrolyte at a temperature of 925 °C for 10 min. The appearance of the sample before the measurements was similar to that previously described in [15]. From the anode side, a platinum grid was used as a current collector, which was pressed against the supported anode with spring clamps located in the cold zone of the cell. Before the measurements, the cell cathode was impregnated with a saturated solution of praseodymium nitrate to reduce the polarization resistance of the cathode. The cell was heated with air supplied to both the cathode and the anode channels separated from each other by the sample. At a temperature of 750 °C, the anode was reduced by stepwise replacement of air with argon and then argon with wet hydrogen. An air pump was used to supply air at a rate of 10 L/h to the fuel cell cathode. To create and supply wet gas mixtures of hydrogen and argon in the anode channel of the fuel cell, high-purity compressed (bottled) gases and mass-flow controllers (Bronkhorst, Bronckhorst, The Netherlands) were used. All the measurements were carried out at the atmospheric pressure. The electrochemical performance of the cell was studied by means of impedance spectroscopy using an FRA-1260 and an EI-1287 (Solartron, Ametek, Berwyn, PA, USA). The polarization resistance of the fuel cell was calculated as the difference between the low- and high-frequency intersection of the impedance spectrum with the Real Z axis at the Nyquist plot divided by the cathode area.

## 3. Results

### 3.1. Characterization of the Initial Electrolyte Powder Materials and Suspension Preparation

**Figure 1:**
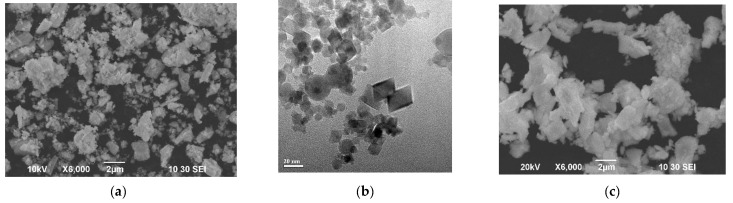
Morphology of the initial electrolyte powders used for the suspension preparation: (**a**) SDC-gn (T_calc_ = 900 °C) (SEM image); (**b**) SDC-lec (TEM image); (**c**) BCS-CuO (T_calc_ = 1150 °C) (SEM image).

The results of the XRD characterization of the SDC-gn, SDC-lec and BCS-CuO electrolyte materials are represented in Table 1. All the electrolyte materials were single phase, a fluorite-type structure was exhibited for the SDC powders and a perovskite-type structure was exhibited for the BCS-CuO powder. The morphology of the initial powders used for the suspension preparation is shown in Figure 1. Micro-sized SDC-gn (Figure 1a) and BCS-CuO (Figure 1c) powders are characterized by the presence of irregularly shaped particles of 1–3 μm in size, as well as their aggregates, including those containing smaller submicron particles. The particles of the SDC-lec nano-sized powder (Figure 1b) were of both spherical and faceted shapes (average size of 9 nm), which may be due to the nature of the particle condensation during their synthesis from a vapor–gas mixture during the growth of crystal faces on solid nucleation particles. The specific surface area of the SDC-gn, SDC-lec and BCS-CuO powders was around 12, 44 and 3 m^2^/g, respectively.

As mentioned in the Materials and Methods section, the suspensions were prepared in a mixed isopropanol/acetylacetone (70/30 vol.%) dispersion medium. The results of the electrokinetic studies (measurement of the zeta potential), performed in the SDC-gn suspension with a concentration of 10 g/L with different durations of ultrasonic treatment (UT) of 5–125 min, are presented in Table 2. 

Modification of the suspension of the micro-sized SDC-gn powder was carried out by adding the SDC-lec nanopowder in an amount of 5 wt%. According to the results obtained, the zeta potential value of the studied suspensions reached a stable value after 25 min of ultrasonication, while the pH shifted to the acidic side. The addition of the SDC-lec nanopowder allowed the zeta potential of the resulting composite suspension to be increased to +13 mV without the introduction of any dispersants or charging agents. The addition of SDC-lec was found to increase the stability of the composite suspension and its zeta potential, probably due to the formation of a “cloud” of SDC-lec nanoparticles diffusely distributed around micro-sized particles of the main SDC-gn powder, which prevents aggregation and allows the stable EPD process to be carried out. 

The suspension of the micro-sized BCS-CuO powder with a concentration of 10 g/L was sonicated for 125 min. The zeta potential was measured in both the initial BCS-CuO suspension and that prepared with the addition of iodine in an amount of 0.4 g/L. Molecular iodine was added to the suspension as a charging agent to increase the efficiency of the EPD, as, according to the preliminary experiments, deposition without iodine did not occur. The influence of the addition of molecular iodine on the properties of non-aqueous suspensions of various oxide materials is described in [45,46,47]. The zeta potential and pH values measured for the base BCS-CuO suspension were +11 mV and 4.3, respectively. The zeta potential value in the suspension of BCS-CuO with iodine did not change and amounted to +11 mV; however, the pH value shifted to a more acidic side (pH = 3.7). The addition of iodine probably promotes the generation of protons in the suspension by the reaction with acetylacetone. The resulting composite suspension of 10 g/L SDC-gn + 5 wt% SDC-lec (SDC-gn/lec) and the modified suspension of 10 g/L BCS-CuO with the addition of iodine 0.4 g/L are suitable for the implementation of a stable EPD process. 

### 3.2. Peculiarities of EPD from the Suspensions of the Electrolyte Materials on a Model Ni-foil Substrate 

**Figure 2:**
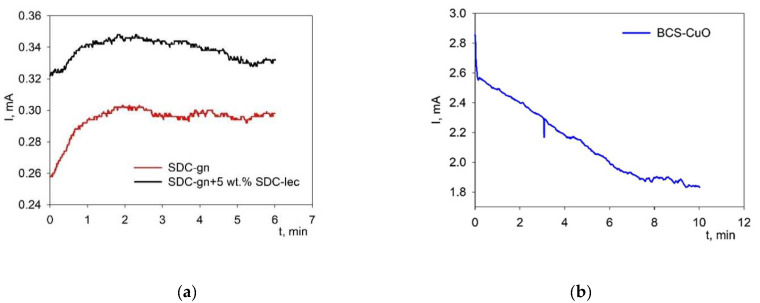
Change in the current strength with time during EPD on the Ni-foil model electrode: (**a**) for the suspensions of 10 g/L SDC-gn/lec and 10 g/L SDC-gn (V = 60 V, t = 6 min); (**b**) for the suspension of 10 g/L BCS-CuO (V = 80 V, t = 10 min).

To establish modes appropriate for the deposition of continuous, crack- and pore-free electrolyte films of the required thickness, studies of the kinetics of the EPD process were carried out on a model Ni-foil electrode. The dependences of the current strength on time (Figure 2) were obtained for the suspensions of 10 g/L of SDC-gn/lec, 10 g/L of SDC-gn (voltage 60 V) and 10 g/L of BCS-CuO (voltage 80 V) in a mixed dispersion medium of isopropanol/acetylacetone (70/30 vol.%). As can be seen from Figure 2a, the change in current with time during the EPD process from the suspensions of SDC-gn/lec and SDC-gn has a similar character, i.e., there is an initial increase in the current strength with its further stabilization. The addition of the SDC-lec nanopowder led to an increase in the current strength by 14% in the SDC-gn/lec suspension compared to the current strength in the initial SDC-gn suspension. This can be associated with an increase in the zeta potential of the modified SDC-gn/lec suspension (Table 2). 

The current strength behavior on time for the suspension of 10 g/L BCS-CuO (Figure 2b) differs from that of the SDC-gn-based suspensions, showing a decrease in the current strength during the EPD process. The current value in the BCS-CuO suspension significantly exceeded that for the suspensions based on SDC-gn, which is probably due to the addition of iodine. 

**Figure 3:**
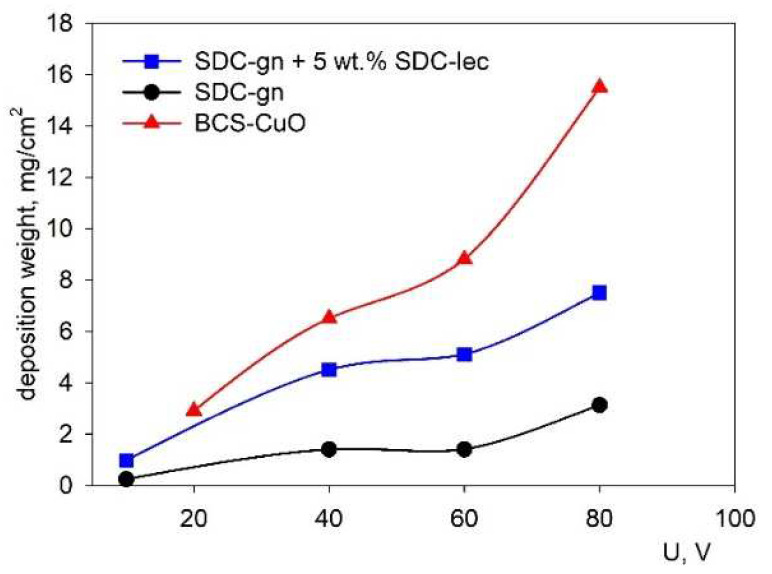
Dependences in the specific weight of the deposited films on the applied EPD voltage for the SDC-gn/lec, SDC-gn and BCS-CuO suspensions (at a constant deposition time of 1 min).

The dependences of the current strength on voltage (current–voltage characteristic (CVC)) obtained for all the suspensions of SDC-gn/lec, SDC-gn and BCS-CuO had a linear character with a change in voltage, which indicates the absence of a dependence of the particle mobility on the magnitude of the electric field strength. The dependences of the deposition weight on voltage (from 10 V to 80 V) measured at a fixed deposition time of 1 min are shown in Figure 3. The dependences obtained were characterized by a non-linear weight increase with increasing voltage. The EPD of the coatings from the composite SDC-gn/lec suspension was accompanied by a large increase in the deposited weight compared to the base SDC-gn suspension. The weight of the deposited coatings during EPD from the BCS-CuO suspension was significantly higher compared to that of the films formed in the SDC-gn suspensions, which is consistent with the above discussed measurement results on the current kinetics versus time (Figure 2). 

**Figure 4:**
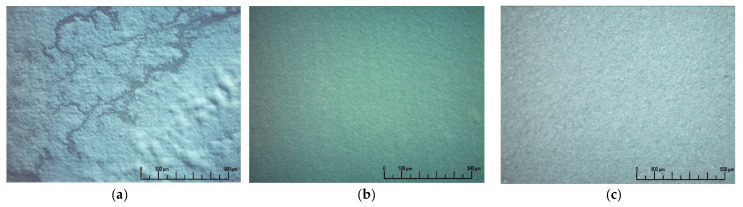
Optical images of the green EPD coatings deposited on the Ni-foil substrate (80 V, 1 min): (**a**) SDC-gn; (**b**) SDC-gn/lec; (**c**) BCS-CuO.

The morphology of the green EPD coatings deposited on the Ni-foil substrate (80 V, 1 min) is shown in Figure 4. 

When using the SDC-gn base suspension, the formation of a continuous coating did not occur (Figure 4a), while the modification of the base suspension with the addition of 5 wt% SDC-lec nanopowder allowed the formation of a continuous, crack-free coating (Figure 4b). The EPD process with iodine-modified BCS-CuO suspensions makes it possible to obtain a continuous coating without cracks (Figure 4c). As we noted above, without the addition of iodine the EPD process did not occur (Section 3.1).

**Figure 5:**
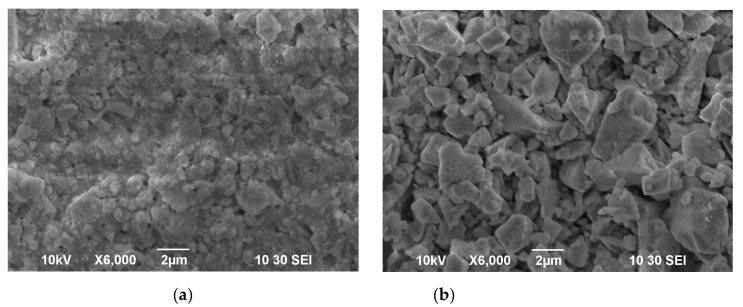
SEM images of the green EPD coatings deposited on the Ni-foil substrate (80 V, 1 min): (**a**) SDC-gn/lec; (**b**) BCS-CuO.

SEM images of the green EPD coatings deposited at 80 V for 1 min from the composite suspension of SDC-gn/lec and the BCS-CuO suspension modified with iodine are shown in Figure 5. For the coating obtained from the SDC-gn/lec suspension, smaller particles filled the gaps between large micro-sized SDC-gn particles that formed the coating. The thickness of the resulting coating was 11 μm. The 24 µm thick coating obtained from the suspension of micro-sized BCS-CuO powder (Figure 5b) comprised ~1–3 µm particles, which is consistent with the morphology of the initial BCS-CuO powder (Figure 1c).

The preliminary studies of the suspensions and experiments on EPD on the Ni-foil substrate made it possible to determine the necessary options for modifying suspensions that allow the stable EPD process to be carried out. The essence of suspension modification consisted in adding the SDC-lec nanopowder in an amount of 5 wt% to the SDC-gn base suspension, as well as adding 0.4 g/L of molecular iodine to the BCS-CuO base suspension. 

### 3.3. Dilatometric Studies of the Sintering Kinetics

**Figure 6:**
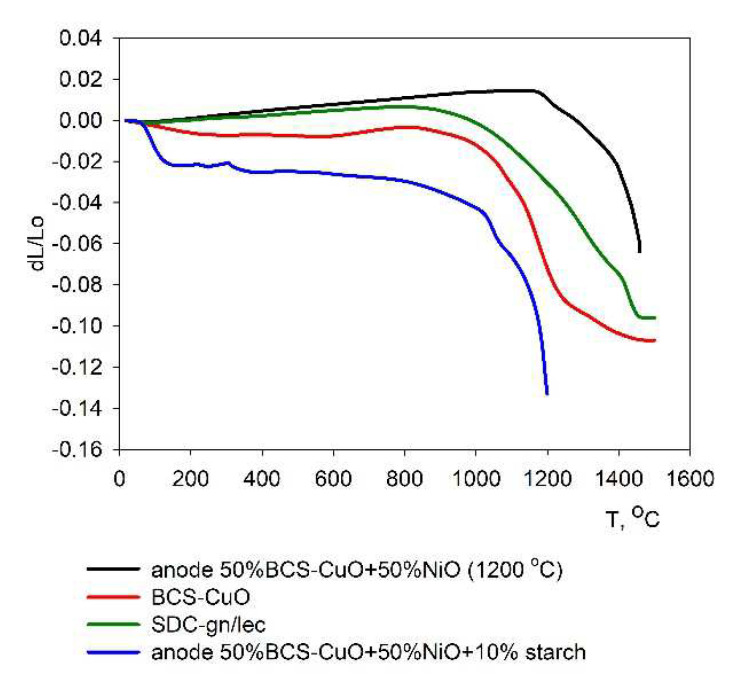
Dilatometric shrinkage curves of the SDC-gn/lec and BCS-CuO electrolyte materials, the 50 wt% NiO + 50 wt% BCS-CuO + 10 wt% starch anode material and the 50 wt% NiO + 50 wt% BCS-CuO anode material, pre-sintered at 1200 °C.

To justify the compatibility of the anode substrate and the electrolyte films’ shrinkage during the sintering, the electrolyte materials SDC-gn/lec, BCS-CuO and an anode substrate made from a mixture of 50 wt% NiO + 50 wt% BCS-CuO + 10 wt% starch pre-sintered at a temperature of 1200 °C were studied using a high-temperature dilatometry method. The results are represented in Figure 6. The unsintered anode material 50 wt% NiO + 50 wt% BCS-CuO + 10 wt% starch had a significant shrinkage, which substantially exceeded the shrinkage of the SDC-gn/lec and BCS-CuO electrolyte materials. In this regard, it was more preferable to carry out preliminary sintering of anodes at a temperature of 1200 °C. It can be seen that the NiO-BCS-CuO anode substrate, pre-sintered at 1200 °C, under the second round of heating began to sinter from a temperature of 1200 °C, the shrinkage rate accelerated sharply when it approached a temperature of 1500 °C and the shrinkage value of the anode sample approached the shrinkage value of the SDC-gn/lec and BCS-CuO electrolyte samples, which, in turn, had a similar shrinkage pattern to each other. In general, it can be concluded that the electrolyte materials under consideration and the anode substrate with a preliminary sintering temperature of 1200 °C are satisfactorily compatible during the formation of the SOFC structure (bilayer electrolyte membrane SDC/BCS-CuO/anode NiO-BCS-CuO). It should be noted that the anode substrate pre-sintered at 1200 °C exhibited satisfactory mechanical strength for further manipulations on the cell fabrication compared to the substrates sintered at 1100 °C for 4 h, which has been established experimentally.

### 3.4. Direct EPD from the SDC-gn/lec and BCS-CuO Suspensions on the Porous NiO-BCS-CuO Anode Substrates, Pre-Sintered at 1200 °C

In a number of works by Besra et al. [24,26] and Matsuda et al. [25,32], a variant of EPD on non-conductive substrates is presented, in which particles are deposited from a suspension on the front side of such a substrate under the action of electric field electrodes, one of which is placed on the reverse side of the substrate. The mechanism of deposition, which is implemented under these conditions, is the formation of conductive channels connecting the deposited layer with the reverse side of the substrate and the electrode [24]. The authors have revealed that the voltage applied during EPD depends both on the porosity of the substrate and on the method of attachment of the substrate to the cell electrode [24] and can vary over a wide range, from 100 to 600 V. The authors of the above works carried out deposition on substrates, sintered at 1000 °C with open porosity, the value of which reached 60% and above. However, manipulations with highly porous substrates are complicated due to their fragility. An analysis of the factors influencing direct deposition has shown that deposition is possible both on substrates with open and closed porosity [23]. In our first experiments on direct deposition, we used NiO-SDC anode substrates fabricated without a pore former during EPD of SDC and BCZYYbO-CuO electrolyte materials in the deposition mode of 250 V for 3 min [33]. The need to increase the voltage during direct deposition on porous substrates, in contrast to the deposition on a model electrode, is due to the fact that the charge transfer process is hindered by the presence of a non-conductive layer located between the deposited layer and the electrode. Despite the high applied voltage, nevertheless, in our previous work, it was not possible to obtain a uniform continuous electrolyte layer, which was, possibly, due to the low porosity of the substrates and its uneven distribution. 

In this work, 50 wt% NiO + 50 wt% BCS-CuO substrates with a pore former (10 wt% of starch) were used, the porosity of which reached 41% after sintering at 1200 °C for 4 h. Direct EPD of the first layer was carried out from the suspension of 10 g/L BCS-CuO in a deposition mode of 200 V for 40 s; the thickness of the green BCS-CuO layer was 13 μm. The second layer was deposited from a suspension of 10 g/L SDC-gn/lec in a deposition mode of 200 V for 60 s; the thickness of the green SDC layer was 10 μm. The layers were dried at room temperature in a Petri dish for 24 h. Two samples with a two-layer BCS-CuO/SDC electrolyte on a NiO-BCS-CuO substrate (BCS-1 and BCS-2) were fabricated with the same modes of deposition of the electrolyte layers for the subsequent selection of an optimal sintering mode. During sintering, the samples with a two-layer BCS-CuO/SDC electrolyte were placed on a platinum plate and covered with an alundum crucible to prevent Ba evaporation. Sintering was carried out at temperatures of 1550 °C and 1500 °C with the same isothermal holding for 5 h. Surface images for the BCS-1 are presented in Figure S1. On the BCS-1 sample, the grains and the intergranular phase were enriched with barium (Figure S1) with predominant migration of barium into the intergranular phase, probably due to diffusion redistribution during high-temperature sintering (1550 °C) and its activation due to the addition of copper to the BCS-CuO electrolyte material and the NiO-BCS-CuO anode substrate. As a result, a continuous dense composite electrolyte membrane was formed, consisting of grains with an average size of ~15 μm. The sintering temperature was too high, and the samples were partly fused to the substrate. Reducing the sintering temperature to 1500 °C for the BCS-2 sample (Figure S2) led to a decrease in barium segregation at the grain boundaries; the average grain size did not change. From the fracture images shown in Figure S3, the BCS-2 film thickness was defined to be equal at approximately 20 µm. The films of BCS and SDC were not distinguished on the fracture images. Moreover, the surface analysis showed a uniform distribution of Ba across the surface of the SDC film. Thus, the film was more of a BCS-SDC composite as a bilayer BCS/SDC electrolyte. It is interesting to note that our results are close to those obtained in previous studies concerning the synthesis and investigation of the properties of the composite electrolytes based on doped BaCeO_3_ and CeO_2_ [48,49]. It was shown that such compositions can be successfully obtained using a one-pot synthesis with the following registration of both phases on the XRD diagrams. However, the SEM studies performed in these studies demonstrated that all the composite elements were uniformly distributed over the sample volume and no individual grains with a micron size related to the phase of barium cerate or cerium oxide were observed. Thus, the authors concluded that the distribution of the two phases was not at the micro level, as it was in [50], but at the submicron level. Similarly, during the stepwise EPD of green films we had the same result. The sintering temperature of the composites in these studies was 1500 °C, which is close to the conditions used in the present study. However, the presence of Cu in the electrolyte and anode could facilitate the mixing of the phases even more.

**Figure 7:**
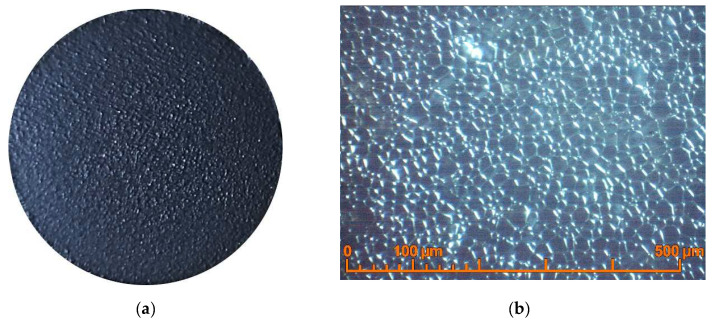
Surface of the BCS-CuO/SDC electrolyte layer after sintering at a temperature of 1450 °C for 5 h on the NiO-BCS-CuO anode substrate (BCS-3 sample): (**a**) the sample appearance (photo image without enlargement); (**b**) optical image.

The experiments on the sintering of the BCS-1 and BCS-2 samples showed the necessity to further reduce the sintering temperature of the BCS-CuO/SDC electrolyte on the NiO-BCS-CuO anode substrate to prevent diffusion and redistribution of barium in the electrolyte membrane. The BCS-3 sample was fabricated by direct EPD with a BCS-CuO green layer thickness of ~18 µm. The deposition of the BCS-CuO layer was carried out in the mode of 200 V for 60 s. Further, the direct EPD of the SDC layer was carried out in the mode of 200 V for 60 s; the thickness of the obtained green SDC layer was 10 μm. The layers were dried at room temperature in a Petri dish for 24 h. Sintering was carried out at a temperature of 1450 °C for 5 h. As a result, according to the optical microscopy data, after sintering, a dense, crack-free coating with a grained surface structure was formed (Figure 7). 

**Figure 8:**
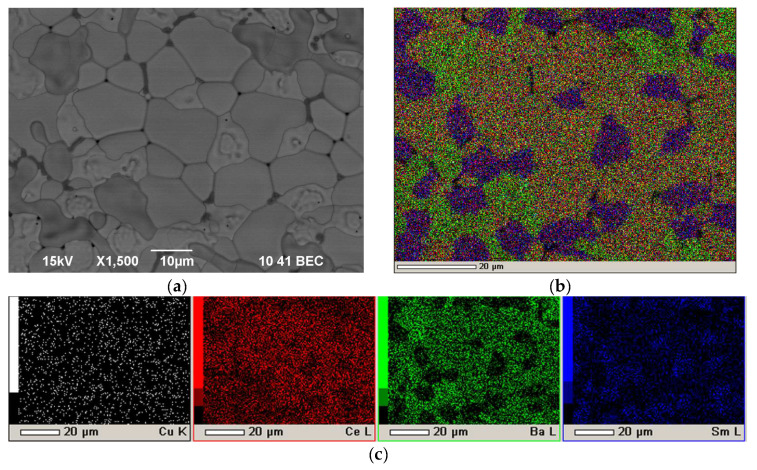
Surface of the BCS-CuO/SDC electrolyte layer after sintering at a temperature of 1450 °C for 5 h on the NiO-BCS-CuO anode substrate (BCS-4 sample): (**a**) SEM image, magnification ×1500; (**b**) integral map of the elements’ distribution; (**c**) individual elements’ maps.

To determine the effect of the ratio between the thicknesses of the BCS-CuO blocking layer and the main SDC layer on the cell performance, the BCS-4 sample was fabricated by direct EPD with the BCS-CuO layer thickness of ~13 μm. The deposition of the BCS-CuO layer was carried out in the mode of 200 V for 40 s. Further, the EPD of the SDC layer was carried out in the mode of 200 V for 110 s; the thickness of the obtained green SDC layer was 18 μm. The layers were dried at room temperature in a Petri dish for 24 h followed by sintering at 1450 °C for 5 h. As a result, according to the optical microscopy data, after sintering, a dense uniform coating with a grainy surface structure was formed, similar to that of BCS-3. The BCS-4 sample microstructure is characterized by the formation of a dense grain structure of the BCS-CuO/SDC composite electrolyte with grain sizes up to 15 µm (Figure 8).

The surface of the electrolyte films sintered at different temperatures was analyzed using the XRD method. To define the main and secondary phases, the computer program QualX2.0/QualX for the qualitative phase analysis of the powder diffraction data was used [51,52]. The results are presented in Figures S4–S6. The main phase detected for the samples sintered at 1550 and 1500 °C was BaCeO_3_ in an amount of approximately 95% and 84%, respectively (Figures S4 and S5). The amount of Sm-doped CeO_2_ was identified to be equal to ~2%. The appearance of the secondary phase of BaNiSm_2_O_5_ was detected in both samples; however, when decreasing the sintering temperature, it increased from 2.9% to 7.6 % and, additionally, the appearance of complex oxide of Ba_2.7_Ce_1.3_Cu_5.9_O_17.8_Sm_4_ (0.8 %) was registered in the sample sintered at 1500 °C. For the sample sintered at 1450 °C both Sm-doped CeO_2_ and BaCeO_3_ were identified in the amount close to the weight ratio of the films. The secondary phases identified for the latter sample were mainly in the form of BaO, Sm_2_O_3_ and NiO (Figure S6). It can be supposed that at a lower temperature, secondary phases are formed in the form of oxides; upon further heating, they dissolve in the main phase and pass into complex compounds, for example, BaNiSm_2_O_5_. It is possible that the barium cerate phase is more stable at higher temperatures, in comparison to the Sm-doped CeO_2_ phase and detected secondary phases. 

As NiO-BCS-CuO anode substrates containing copper in the composition are characterized by significant shrinkage and sintering, which decreases their porosity and may cause gas-diffusion limitations in the anode, for the sake of comparison, the BCS-5 sample was formed on the anode substrate made from 50 wt% NiO + 50 wt% BCS + 20 wt% starch and pre-sintered at 1200 °C for 4 h. The green layer thicknesses were ~20 µm for BCS-CuO (200 V, 90 s) and ~10 µm for SDC (200 V, 60 s), which are close to those for BCS-3. The layers were dried at room temperature in a Petri dish for 24 h, and the sintering was carried out at a temperature of 1450 °C for 5 h. As a result, after sintering, a dense coating with a grainy surface structure was formed, without cracks and pores, similar to that of BCS-3.

### 3.5. SOFCs’ Testing

**Figure 9:**
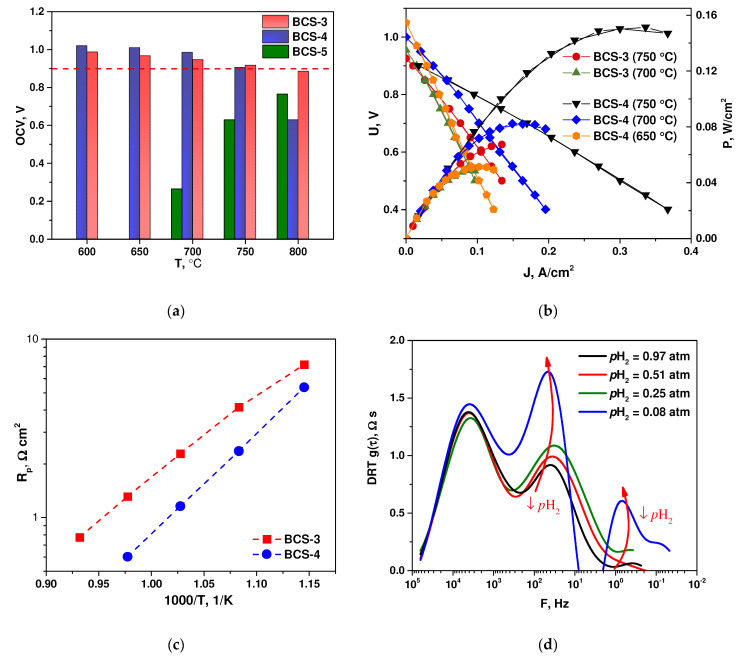
Electrochemical performance of the Ni-BCS-CuO|BCS-CuO/SDC|Pt single cells: (**a**) open circuit voltage (OCV) values of fuel cells at different temperatures; (**b**) volt-ampere and power dependences of the cells at different temperatures; (**c**) temperature dependences of the polarization resistance of the cells’ electrodes; (**d**) frequency dependences of the distribution function of relaxation times for the BCS-4 cell at various partial pressures of hydrogen in the anode channel at 700 °C.

Based on the BCS-3, BCS-4 and BCS-5 half-elements, the single fuel cells were fabricated and their characteristics were studied in the SOFC mode. A model Pt electrode impregnated with praseodymium oxide was used as a cathode of the fuel cells. In the open circuit mode, the BCS-3 and BCS-4 cells showed satisfactory open circuit voltage values in a range of 1.05–0.95 V at temperatures of 600–700 °C (Figure 9a). This is remarkably higher than the OCV values for the SDC film obtained by EPD on the NiO-SDC anode substrate in [53], which were of approximately 0.7 V at 600–700 °C. Moreover, they were slightly higher than the OCV value obtained for a BCS-SDC (1:1 wt. ratio) electrolyte membrane (approximately 30 µm) on the NiO-BCS-SDC anode (0.99–0.94 V at 600–700 °C) [50]. This clearly demonstrates the BCS electronic current blocking influence and validates EPD as a suitable method to obtain composite electrolytes. At 800 °C, however, the open circuit voltage was lower than 0.9 V; thus, it is recommended to use these cells in the IT-SOFC conditions.

It should also be noted that the different behavior of the BCS-5 element, i.e., low OCV values, was apparently caused by insufficient film sintering due to the absence of copper in the carrying BCS-NiO substrate. For this reason, further studies were performed on the BCS-3 and BCS-4 samples. It was found that with increasing temperature, the specific power of the fuel cells increased and amounted to approximately 70 and 50 mWcm^−2^ at 750 and 700 °C, respectively, for BCS-3 and approximately 160, 80 and 50 mWcm^−2^ at 750, 700 and 650 °C, respectively, for BCS-4 (Figure 9b). Such a difference in the specific power obtained for the cells with the close total electrolyte thickness can be due to various reasons. The first possible reason is that the supporting anode of the BCS-4 sample was activated by the impregnation of finely dispersed cerium oxide to reduce its polarization resistance [54]. The effect of cerium oxide on the polarization resistance of the fuel cell is shown in Figure 9c; it can be seen that in the case of the BCS-4 sample the total polarization resistance of the electrodes is much lower due to a decrease in the contribution of the anode resistance (the cathode was the same for both cells). Variation of the partial pressure of hydrogen at the anode of the BCS-4 fuel cell with subsequent analysis of the electrochemical impedance spectra by the distribution of relaxation times (DRT) showed that the resistance of the high-frequency stage did not depend on the partial pressure of hydrogen (Figure 9d). This indicates that this step is associated with an oxygen reduction reaction at the cathode of the fuel cell. The behavior of the mid-frequency and low-frequency processes depend on the partial pressure of hydrogen, which clearly indicates that these stages belong to the hydrogen oxidation reaction at the anode of the cell. The obtained data correlate well with the results obtained in a detailed analysis of the behavior of traditional fuel cells with an oxygen-conducting electrolyte using the DRT method [55]. 

Another possible reason for the increase in the power of the BCS-4 cell is the change in the ratio of the thicknesses of the deposited BCS and SDC films, which in the case of BCS-3 and BCS-4 was 18/10 and 13/18, respectively, and corresponds to the mass ratio of 1.6/1 and 0.64/1. The serial resistance, R_s_, which includes the ohmic resistance of the electrolyte and the resistance of the electrode–electrolyte contact, decreased from 1.23, 1.41, 1.91 and 2.32 Ωcm^2^ for BCS-3 to 0.52, 0.81, 1.19 and 1.66 Ωcm^2^ for BCS-4 at 750, 700, 650 and 600 °C, respectively. The reason for that can be the fact that an increase in the SDC content in the composite leads, as a rule, to an increase in its conductivity [56]. However, as can be seen from Figure 9b, increasing the SDC content results in a decrease of the OCV value. In the case of BCS-3 and BCS-4 it was insignificant—0.92 and 0.90 V, respectively, at 750 °C; however, further increasing the SDC content may result in a remarkable drop in OCV. Nevertheless, in this study the 13/18 ratio in the BCS/SDC film thickness can be considered an optimal one. The comparison of the impedance spectra of the BCS-3 and BCS-4 cells collected at 700 °C (Figure S7) showed that both the polarization and series resistances for the BCS-3 cell was worse than for the BCS-4 cell. It can be seen from Figure S7 that the total resistances of the cells differ by approximately two times, which corresponds to the difference in the power of the fuel cells shown in Figure 9.

Despite the activation of both the anode and cathode, the total polarization resistance values, R_p_, for the BCS-4 cell were 1.15, 2.35 and 5.36 Ωcm^2^ at 700, 650 and 600 °C, respectively, which are much higher than, for example, 0.076, 0.212 and 0.552 Ωcm^2^ obtained in [50] for the BCS-SDC cell with the NiO-BCS-SDC anode and highly active Sm_0.5_Sr_0.5_CoO_3-δ_-SDC cathode. Moreover, R_p_ values exceeded the values of ohmic resistance, thus contributing the most to a decrease in the cell performance, especially at low temperatures. It can be related to the high resistance of the Pt cathode in contact with the Ba-containing phase in the composite electrolyte obtained in this study. A similar increased resistance of the Pt cathode in contact with doped BaCeO_3_ was observed in [57]. Thus, further work should be directed not only on the improvement of the supporting anode content and microstructure but also to the search for relevant MIEC [58] or triple-conducting [59] electrodes that are more active at decreased temperatures compared to platinum. 

**Figure 10:**
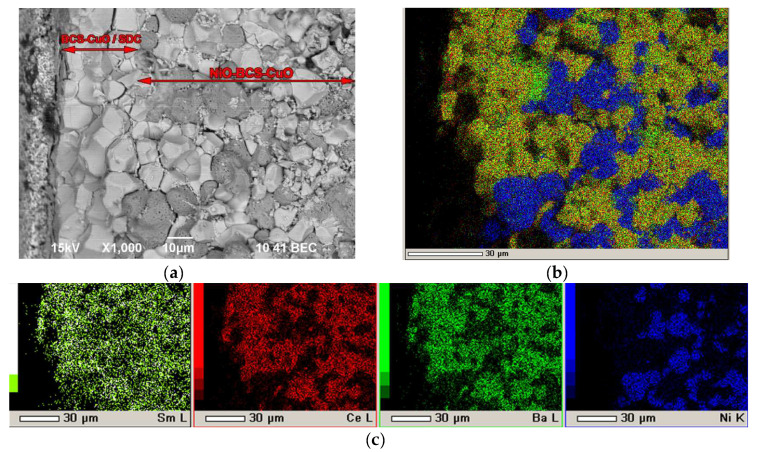
Fracture images of the BCS-4 sample with the BCS-CuO/SDC electrolyte layer on the NiO-BCS-CuO anode substrate (sintered at a temperature of 1450 °C for 5 h) after electrochemical testing in the SOFC mode: (**a**) SEM image, magnification 1500× (**b**) integral map of the elements’ distribution; (**c**) individual elements’ maps.

Microstructure studies were carried out for the BCS-4 sample (Figure 10) after testing in the SOFC mode. As can be seen from Figure 10, the electrolyte membrane with a thickness of approximately 30 µm has a dense grain structure with a grain size of ~10 µm. The distribution of barium and cerium, according to the EDX analysis, is uniform within the film, which indicates the formation of a composite structure of the electrolyte membrane. The electrolyte membrane kept its integrity and good adhesion to the anode substrate after its reduction followed by testing the cell over two days. 

## 4. Conclusions

In this work, the formation of the single SOFC cells with the thin-film BCS-CuO/SDC composite membranes was performed on the NiO-BCS-based porous anode substrates by direct electrophoretic deposition. The electrokinetic properties of the suspensions based on the electrolyte powders of BCS-CuO and SDC materials were studied. A method was proposed for modifying the suspension of micro-sized SDC-gn powder by adding SDC-lec nanopowder (5 wt%). It was shown that the modified SDC-gn/lec suspension exhibited a higher zeta potential value relative to that of the base SDC-gn suspension, which improved the uniformity of the deposited SDC films. It was shown that the deposition from the BCS-CuO suspensions required the addition of molecular iodine (0.4 g/L), which, however, did not change the zeta potential value but initiated the EPD process. The dependences of the current on time for all the suspensions during the EPD process were studied. The dependences of the deposition weight on voltage were obtained for a fixed deposition time on a Ni-foil model electrode. The morphology of the green EPD coatings was studied. It was shown that the green coating on SDC-gn/lec consisted of micro-sized particles, the gaps between which were filled with submicron particles; the BCS-CuO green coating was formed by micro-sized particles, the morphology of which corresponded to the original powder. A feature of this work was that the direct EPD of the SDC-gn/lec and BCS-CuO electrolyte materials was performed on the NiO-BCS-based porous anode substrates pre-sintered at a temperature of 1200 °C for 4 h (porosity 41%). It was found that the porosity of the used NiO-BCS substrates was sufficient for the implementation of sequential direct EPD of the bilayer BCS-CuO/SDC electrolyte. The characteristics of sintering and shrinkage of the electrolyte materials and the anode substrate material were studied. The compatibility of the materials used made it possible to obtain the dense composite electrolyte membrane at a temperature of 1450 °C for 5 h. The electrochemical study of the cells revealed high OCV values and the post-test cells’ microstructural characterization confirmed the gas-tightness of the electrolyte films. Analysis of the obtained data in the SOFC mode showed that further work is required to improve the composition and microstructure of electrodes in order to achieve high cell performance. 

## Figures and Tables

**Table 1:** Characteristics of the SDC-gn, SDC-lec and BCS-CuO electrolyte materials.

Electrolyte Material	Crystal Lattice Type, Space Group	Lattice Parameters, Å	Specific Surface Area, m^2^/g
SDC-gn	cubic-type structure, Fm-3m (225)	a = 5.431(1) Å	12.1(2)
SDC-lec	cubic-type structure, Fm-3m (225)	a = 5.439(3) Å, CSR*=18(2) nm	44.1(9)
BCS-CuO	orthorhombic structure, Pnma (62)	a = 6.231(1) Å, b = 8.801(2) Å, c = 6.227(1) Å	3.0(1)

*CSR—coherent scattering region.

**Table 2:** Electrokinetic properties of the base suspension of the SDC-gn powder (10 g/L) and the SDC-gn suspension modified with the addition of SDC-lec nanopowder (5 wt%).

Suspension	UT, min	Zeta Potential, mV (pH)
SDC-gn	5 25 125	+4 (5.9) +6 (4.0) +6 (3.5)
SDC-gn + 5 wt% SDC-lec	5 25	+13 (3.6) +13 (3.6)

## Data Availability

Not applicable.

## Supplementary Materials

The following are available online at https://www.mdpi.com/article/10.3390/membranes12070682/s1, Figure S1: The surface of the BCS-CuO/SDC electrolyte layer after sintering at a temperature of 1550 °C for 5 h on the NiO-BCS-CuO anode substrate (BCS-1 sample): (a) SEM image, magnification 3000× (b) Integral map of the elements’ distribution; (c) Individual elements’ maps, Figure S2: The surface of the BCS-CuO/SDC electrolyte layer after sintering at a temperature of 1500 °C for 5 h on the NiO-BCS-CuO anode substrate (BCS-2 sample): (a) SEM image, magnification 3000× (b) Integral map of the elements’ distribution; (c) Individual elements’ maps, Figure S3: Fracture images of the BCS-2 sample with the BCS-CuO/SDC electrolyte layer on the NiO-BCS-CuO anode substrate after sintering at a temperature of 1500 °C for 5 h: (a) SEM image, magnification 1500× (b) Integral map of the elements’ distribution; (c) Individual elements’ maps, Figure S4: XRD-data for the BCS-1 sample, sintered at 1550 °C, Figure S5: XRD-data for the BCS-2 sample, sintered at 1500 °C, Figure S6: XRD-data for the BCS-4 sample, sintered at 1450 °C, Figure S7: EIS spectra for the BCS-3 and BCS-4 cells collected at 700 °C.
