# Supplementary material for: Direct Electrophoretic Deposition and Characterization of Thin-Film Membranes Based on Doped BaCeO3 and CeO2 for Anode-Supported Solid Oxide Fuel Cells
Source: Membranes (Basel). 2022 Jun 30;12(7):682. doi: 10.3390/membranes12070682 (PMC9316799; doi:10.3390/membranes12070682)
Supplement: Supplementary file 1 [file membranes-12-00682-s001.zip › membranes-1784521-supplementary.pdf]

Supplementary Materials

Direct Electrophoretic Deposition and Characterization of Thin-film Membranes Based on Doped $BaCeO_3$ and CeO_2 for Anode-supported Solid Oxide Fuel Cells

Elena Pikalova[1,2], Denis Osinkin[1,2], Elena Kalinina[3,4*]

[1] Laboratory of Solid Oxide Fuel Cells, Institute of High Temperature Electrochemistry, Ural Branch of the Russian Academy of Sciences, Yekaterinburg 620137, Russia; e.pikalova@list.ru, osinkinda@mail.ru

[2] Department of Environmental Economics, Graduate School of Economics and Management, Ural Federal University, Yekaterinburg 620002, Russia; e.pikalova@list.ru

[3] Laboratory of Complex Electrophysic Investigations, Institute of Electrophysics, Ural Branch of the Russian Academy of Sciences, Yekaterinburg 620016, Russia; jelen456@yandex.ru

[4] Department of Physical and Inorganic Chemistry, Institute of Natural Sciences and Mathematics, Ural Federal University, Yekaterinburg 620002, Russia; jelen456@yandex.ru

* Correspondence: jelen456@yandex.ru (E.K.); Tel.: +7-343-267-87-82 (E.K.)

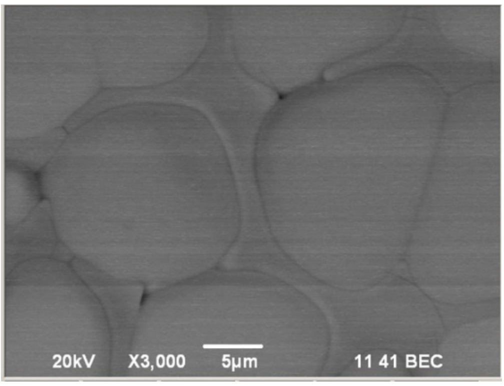

(a)

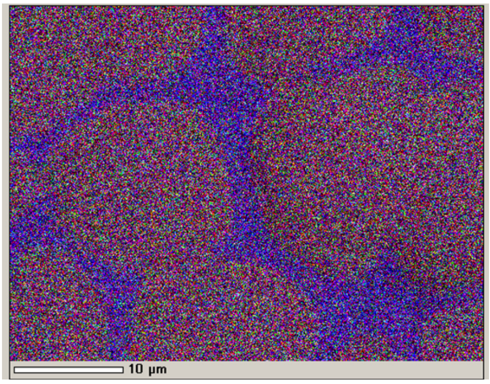

(b)

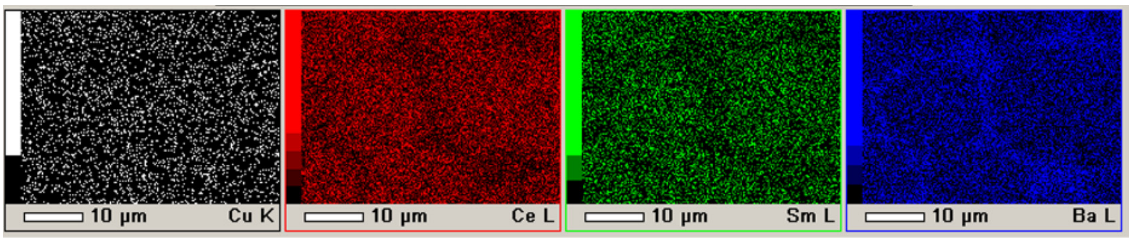

(c)

Figure S1: The surface of the BCS-CuO/SDC electrolyte layer after sintering at a temperature of 1550 °C for 5 h on the NiO-BCS-CuO anode substrate (BCS-1 sample): (a) SEM image, magnification x3000; (b) Integral map of the elements' distribution; (c) Individual elements' maps.

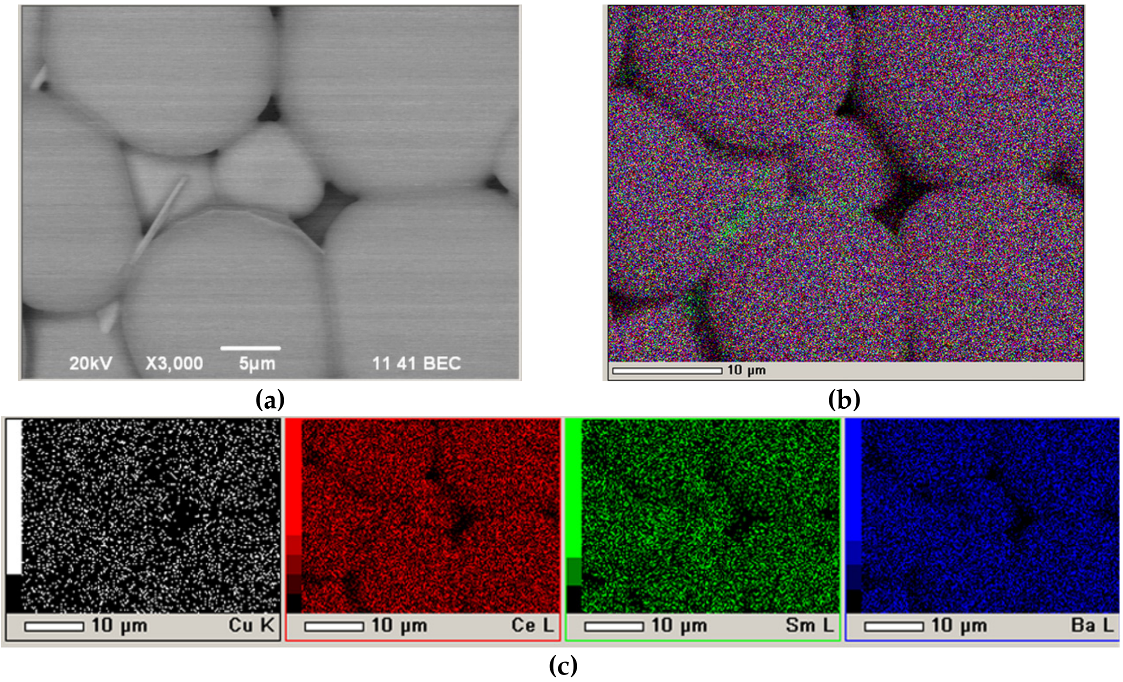

Figure S2: The surface of the BCS-CuO/SDC electrolyte layer after sintering at a temperature of 1500 °C for 5 h on the NiO-BCS-CuO anode substrate (BCS-2 sample): (a) SEM image, magnification x3000; (b) Integral map of the elements' distribution; (c) Individual elements' maps.

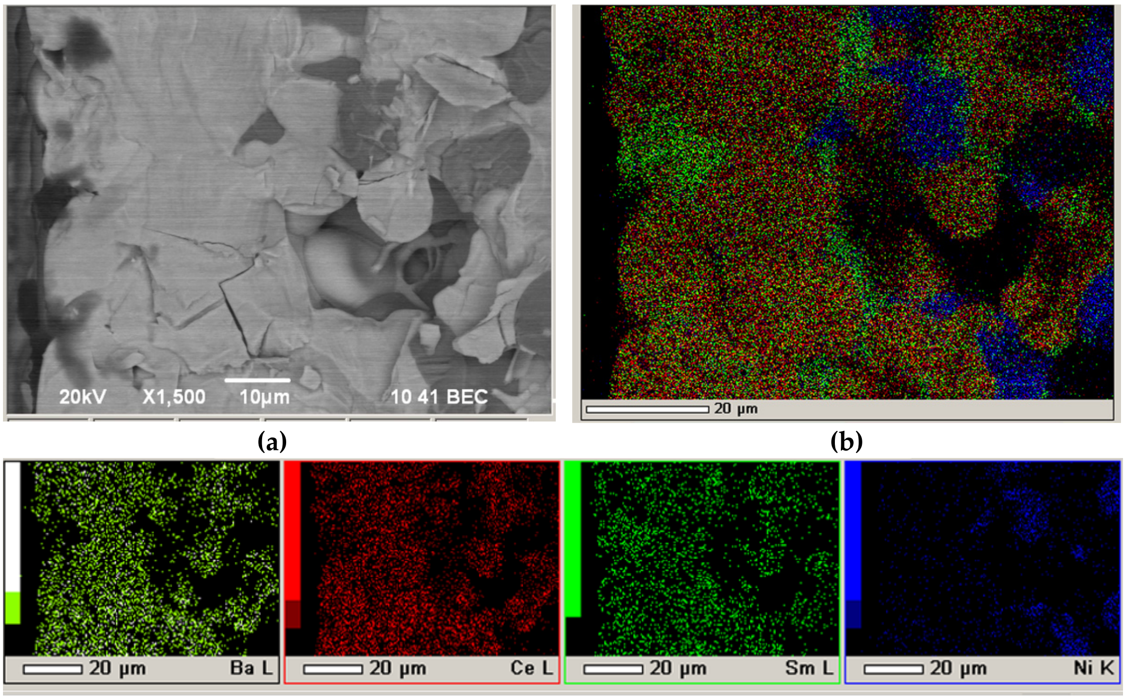

(a) (b)

(c)

Figure S3: Fracture images of the BCS-2 sample with the BCS-CuO/SDC electrolyte layer on the NiO-BCS-CuO anode substrate after sintering at a temperature of 1500 °C for 5 h: (a) SEM image, magnification x1500; (b) Integral map of the elements' distribution; (c) Individual elements' maps.

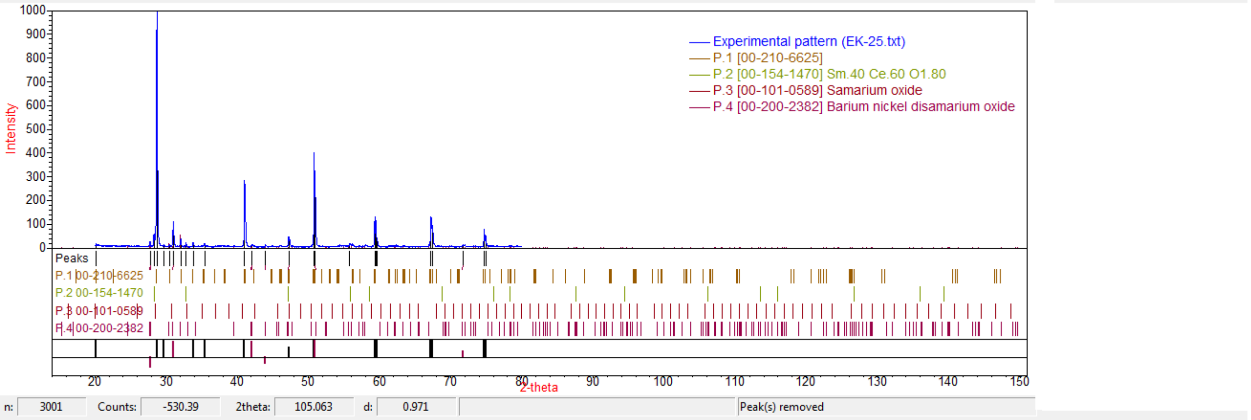

No.	QM	CARD	Compound Name	Chemical Formula	Peakpos.	Intensity	Scale	FoM	S-Quant.
P.1	C	00-210-6625		Ba Ce O3	0.66198	0.21878	0.38084	0.69790	94.7%
P.2	C	00-154-1470	Sm.40 Ce.60 O1.80	Ce0.6 O1.8 Sm0.4	0.70887	0.79362	0.63533E-01	0.64089	2.1%
P.3	C	00-101-0589	Samarium oxide	Sm2 O3	0.74212	0.63472E-01	0.75756E-02	0.29329	0.3%
P.4	C	00-200-2382	Barium nickel disamarium oxide	Ba Ni Sm2 O5	0.48514	0.43646	0.54429E-01	0.52862	2.9%

Figure S4: XRD-data for the BCS-1 sample, sintered at 1550 °C.

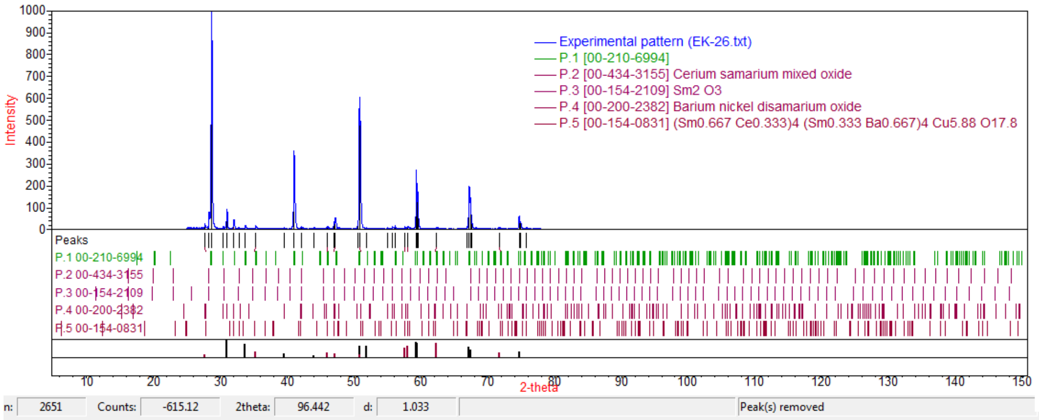

No.	QM	CARD	Compound Name	Chemical Formula	Peakpos.	Intensity	Scale	FoM	S-Quant.
P.1	C	00-210-6994		Ba Ce O3	0.63250	0.39286	0.77312	0.76187	84.5%
P.2	C	00-434-3155	Cerium samarium mixed oxide	Ce0.4 O1.7 Sm0.6	0.75985	0.46810	0.75905E-01	0.67836	6.5%
P.3	C	00-154-2109	Sm2 O3	O3 Sm2	0.58897	0.43912E-01	0.66108E-02	0.31860	0.6%
P.4	C	00-200-2382	Barium nickel disamarium oxide	Ba Ni Sm2 O5	0.60128	0.43874	0.55633E-01	0.63082	7.6%
P.5	C	00-154-0831	(Sm0.667 Ce0.333)4 (Sm0.333 Ba0.667)4 Cu5.88 O17.8	Ba2.668 Ce1.332 Cu5.88 O17.8 Sm4	0.54475	0.39222E-01	0.61808E-02	0.35165	0.8%

Figure S5: XRD-data for the BCS-2 sample, sintered at 1500 °C

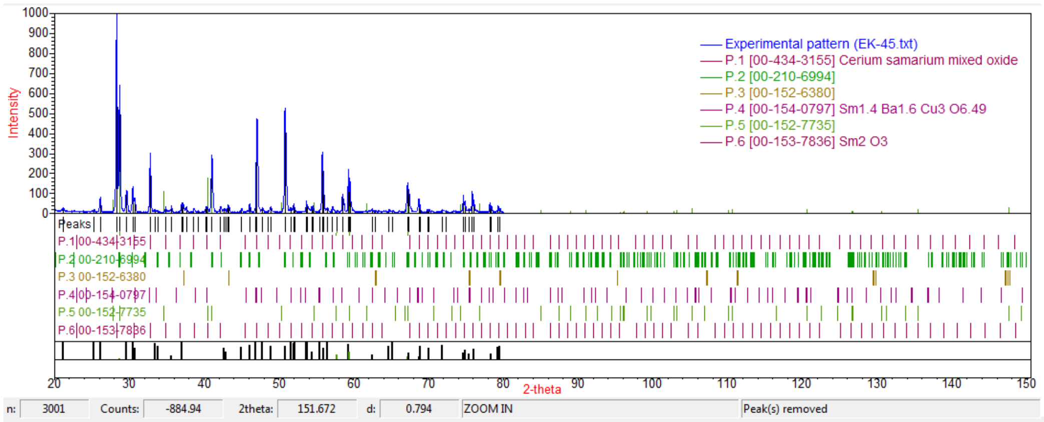

No.	QM	CARD	Compound Name	Chemical Formula	Peakpos.	Intensity	Scale	FoM	S-Quant.
P.1	C	00-434-3155	Cerium samarium mixed oxide	Ce0.4 O1.7 Sm0.6	0.81261	0.58184	0.99531	0.75204	44.1%
P.2	C	00-210-6994		Ba Ce O3	0.64695	0.20096	0.54732	0.67203	30.9%
P.3	C	00-152-6380		Ni O	0.53648	0.60349	0.31263E-01	0.61567	3.8%
P.4	C	00-154-0797	Sm1.4 Ba1.6 Cu3 O6.49	Ba1.6 Cu3 O6.49 Sm1.4	0.48762	0.19452E-01	0.69047E-02	0.33334	0.5%
P.5	C	00-152-7735		Ba O	0.58910	0.32874	0.20911	0.45609	19.2%
P.6	C	00-153-7836	Sm2 O3	O3 Sm2	0.63447	0.11274	0.33410E-01	0.40668	1.5%

Figure S6: XRD-data for the BCS-4 sample, sintered at 1450 °C

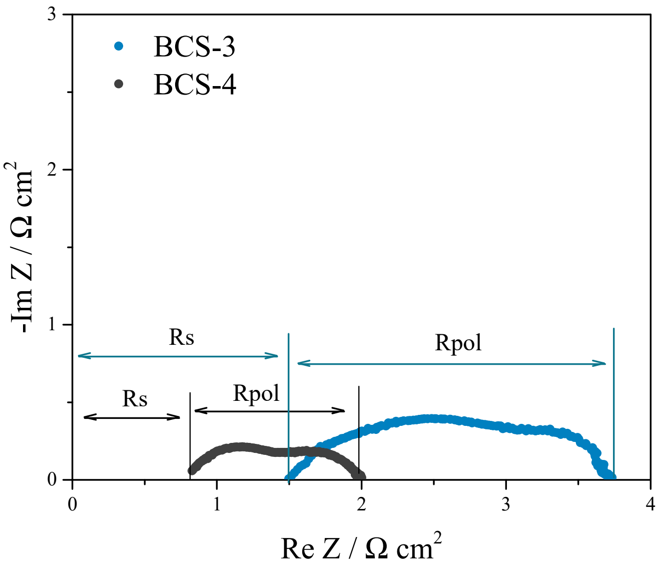

Figure S7: EIS spectra for the BCS-3 and BCS-4 cells collected at 700 °C.
